# Oral Microbiota Analysis of Tissue Pairs and Saliva Samples From Patients With Oral Squamous Cell Carcinoma – A Pilot Study

**DOI:** 10.3389/fmicb.2021.719601

**Published:** 2021-10-12

**Authors:** Ke Yang, Yuezhu Wang, Shizhou Zhang, Dongsheng Zhang, Lihua Hu, Tengda Zhao, Huajun Zheng

**Affiliations:** ^1^Department of Oral and Maxillofacial Surgery, Shandong Provincial Hospital Affiliated to Shandong First Medical University, Jinan, China; ^2^Department of Health Management Center, Shandong Provincial Hospital Affiliated to Shandong First Medical University, Jinan, China; ^3^NHC Key Lab of Reproduction Regulation, Shanghai Institute for Biomedical and Pharmaceutical Technologies, Fudan University, Shanghai, China; ^4^Shanghai-MOST Key Laboratory of Health and Disease Genomics, Chinese National Human Genome Center at Shanghai and Shanghai Institute for Biomedical and Pharmaceutical Technologies, Shanghai, China

**Keywords:** oral microbiota, 16S rRNA, *Rothia mucilaginosa*, *Prevotella intermedia*, oral squamous cell carcinoma

## Abstract

Oral microbiota dysbiosis is associated with the occurrence and progression of oral cancer. To investigate the association between the microbiota and risk of oral squamous cell carcinoma (OSCC), we identified the microbial composition of paired tumor (TT)/normal paracancerous tissues (NPT) and saliva (TS) samples in OSCC patients through 16S rRNA gene sequencing. A total of 22 phyla, 321 genera, and 869 species were identified in the oral samples. Paired comparisons revealed significant differences between TT, NPT, and TS groups, with the genus *Filifactor* significantly enriched in TT. The phylum Actinobacteria; genus *Veillonella*; and species *Granulicatella adiacens*, *Streptococcus sanguinis*, and *Veillonella rogosae* were significantly enriched in NPT, while the phylum Bacteroidetes; genera *Capnocytophaga*, *Haemophilus*, and *Prevotella*; and seven species, including *Capnocytophaga* sp., *Haemophilus* sp., and *Neisseria* sp., were significantly enriched in TS. In TTs, the abundance of *Prevotella intermedia* was profoundly higher in the gingiva, while *Capnocytophaga gingivalis* and *Rothia mucilaginosa* were enriched in the lining mucosa and tongue. Increasing in abundance from the early tumor stage to the late stage, *Solobacterium moorei* in TT and *Campylobacter* sp. *strain HMT 044* in TS were positively correlated with OSCC development, suggesting that bacteria were selected by different microenvironments. The correlation between 11 microbial species and 17 pathway abundances was revealed, indicating the potential function of low-abundance bacteria. Overall, our analysis revealed that multiple oral bacterial taxa are associated with a subsequent risk of OSCC and may be used as biomarkers for risk prediction and intervention in oral cancers.

## Introduction

Annually, more than 447,000 new diagnoses of and nearly 228,000 deaths due to oral cancer are reported worldwide, over 90% of which can be attributed to squamous cell carcinoma (SCC) (Bray et al., 2018). SCC is defined as an epithelial malignancy with poor prognosis that originates from the buccal mucosa, tongue, mouth floor, gingiva, and oropharynx, with a 5-year relative survival rate of less than 65% (Siegel et al., 2020). Environmental triggers, such as tobacco, alcohol consumption, and betel quid chewing, along with human papillomavirus infection, specifically identified in the oropharynx, are major risk factors for oral cancer. In addition, poor oral hygiene and periodontal disease, which affect bacterial ecology, have been epidemiologically and independently linked to oral cancer (Healy and Moran, 2019).

Specific bacteria have been implicated in cancer development. A typical example of a bacterial carcinogen is *Helicobacter pylori*, which is known to promote gastric cancer (Amieva and Peek, 2016). In the human oral cavity, more than 700 different bacterial species have been identified, and dysbiosis of these species results in significant and distant effects in patients with gastrointestinal, hepatobiliary, pancreatic, and colorectal tumors (Mascitti et al., 2019). It has been suggested that the oral microbiota plays an important role in carcinogenesis by stimulating chronic inflammation, producing carcinogenic substances, or inhibiting cell apoptosis (Karpinski, 2019).

Oral bacteria are most likely linked to oral squamous cell carcinoma (OSCC) (Whitmore and Lamont, 2014). Four common inhabitants of the oral cavity, *Porphyromonas gingivalis*, *Fusobacterium nucleatum*, *Treponema denticola*, and *Streptococcus anginosus*, have been identified as potential etiologic agents of OSCC (Zhang W.L. et al., 2019). Bacterial culture demonstrated that significantly higher levels of *Veillonella, Porphyromonas*, and *Fusobacterium* are harbored by tumor surface of OSCC than healthy mucosa (Nagy et al., 1998). Mager et al. (2005) revealed that the levels of three species (*Capnocytophaga gingivalis*, *Prevotella melaninogenica*, and *Streptococcus mitis*) were elevated in the saliva of patients with OSCC. *S. anginosus*, a pathogen that occurs frequently in OSCC tissue specimens, was detected in the dental plaque but not in the saliva of patients (Sasaki et al., 2005). By comparing OSCC tissues and adjacent non-tumor mucosa, Pushalkar et al. (2012) revealed that *Streptococcus* sp. (*S. salivarius*, *S. gordonii*, and *S. parasanguinis*), *Gemella* sp. (*G. haemolysans* and *G. morbillorum*), *Johnsonella ignava*, and *Peptostreptococcus stomatis* were prevalent in tumor sites, whereas *Granulicatella adiacens* was prevalent in non-tumor sites.

With the advent of 16S rRNA high-throughput sequencing, there is growing interest in the possible relationships between changes in the oral microbiota and the risk of oral cancer (Guerrero-Preston et al., 2016). Lee et al. (2017) found that five genera (*Bacillus*, *Enterococcus*, *Parvimonas*, *Peptostreptococcus*, and *Slackia*) exhibited significant differences in saliva between epithelial precursor lesions and OSCC patients, and the genera *Cloacibacillus*, *Gemmiger*, *Oscillospira*, and *Roseburia* were 20 times more abundant in OSCC patients than in healthy controls. Yang et al. compared oral mouthwash samples from OSCC patients and healthy controls and found that the abundances of *Fusobacterium periodonticum* and *Streptococcus constellatus* were positively correlated with the development of OSCC, whereas *S. mitis*, *Haemophilus parainfluenzae*, and *Porphyromonas pasteri* were negatively correlated (Yang et al., 2018). Zhao et al. (2017) and Zhang L. et al. (2019) compared the epithelial samples collected from tumor sites and normal tissues of patients with OSCC using swabs. Zhang L. et al. (2019) found that 10 species (including *F. nucleatum* and *Prevotella intermedia*) and genes involved in bacterial chemotaxis, flagellar assembly, and lipopolysaccharide (LPS) biosynthesis were significantly increased in the tumor sites. Zhao et al. (2017) observed that a group of periodontitis-correlated taxa, including *Fusobacterium* and *Dialister*, was significantly enriched in tumor sites. By comparing fresh OSCC biopsies and epithelial samples of healthy controls collected by swab, Al-Hebshi et al. (2017) reported that some specific species in tumor tissues (TTs), such as *F. nucleatum* and *Pseudomonas aeruginosa*, were related to OSCC. Chang et al. (2019) revealed that *P. gingivalis* and *F. nucleatum* were present at higher levels in cancer tissue than in normal tissues of patients with OSCC and were correlated with subgingival plaques. By comparing OSCC tissue, saliva, and mouthwash samples from the same subjects, Zhang Z. et al. (2019) revealed that the genera *Acinetobacter* and *Campylobacter* were enriched in OSCC tissues, while *Streptococcus* and *Prevotella* were enriched in saliva and mouthwash.

According to a growing number of studies, over 30 genera and a panel of bacterial species have been associated with OSCC (Sami et al., 2020). However, we could see variations in OSCC-associated bacteria among the above-mentioned studies, which might be caused by different bacterial detection techniques and sample types. Traditional methods used in OSCC bacterial studies, such as bacterial culture (Nagy et al., 1998), DNA–DNA hybridization (Mager et al., 2005), PCR (Sasaki et al., 2005), immunohistochemical staining (Katz et al., 2011), and denaturing gradient gel electrophoresis (DGGE) (Pushalkar et al., 2012), usually focus on certain species, while high-throughput sequencing has higher sensitivity. The oral ecosystem has several significantly different niches, including saliva, oral mucosa, and teeth surfaces, each having a unique microbiota (Zhang et al., 2018). A recent study reported that the structure and function of the bacteriome on the surface of oral cancer is significantly different from that within the tumor, suggesting that different types of sample collection (swab, tissue, or saliva) and controls (adjacent mucosa or healthy people) might yield inconsistent conclusions (Gopinath et al., 2021).

To date, comparisons among paired tumor/paracancerous tissues and saliva samples from the same OSCC patients have not been performed. In the current pilot study, we aimed to investigate the association between microbiota and the risk of developing OSCC, as well as to establish the connection between the changes in the microbiota in TT, normal paracancerous tissue (NPT), and saliva (TS). Twenty-three patients with OSCC from Northern China were recruited, and the overall microbiota composition and abundance of specific bacterial taxa in TT, NPT, and TS were identified through 16S rRNA gene sequencing. Furthermore, phylogenetic investigation of communities by reconstruction of unobserved states (PICRUSt2) (Langille et al., 2013) was used to infer the underlying pathways associated with oral cancer. Our study will add new information on OSCC-associated oral microbiota from both saliva and cancer tissue, and provide a target for the diagnosis and treatment of OSCC.

## Materials and Methods

### Subject Enrollment and Sample Collection

The subjects were recruited from 2018 to 2020 with the approval of the Chinese Clinical Trial Registry (ChiCTR2000032543). All participants were not administered any antibiotics 3 months before the study. Written informed consent was obtained from all participants before sample collection. In total, 23 patients with OSCC who had not undergone any treatment, including chemotherapy and/or radiotherapy were enrolled. TNM staging and data on age, sex, alcohol intake, cigarette smoking, oral hygiene, and periodontal status were assessed. Saliva samples were successfully collected from 19 out of the 23 OSCC patients before breakfast without stimulation. Furthermore, TT samples were dissected from the deep site of the tumor during surgery, and the diameter of each sample was larger than 3.0 mm. Concurrently, paired NPT samples harvested at least 2.0 cm away from the tumor edge were also obtained under aseptic conditions. Finally, saliva and tissue samples were aseptically transferred into a screw-cap vial and stored at −80°C immediately after collection.

To characterize the differences in the microbiota composition in different tissue and saliva samples, 65 samples were classified into three groups as follows: TT, NPT, and TS.

### Genomic DNA Extraction, PCR, and 16S rRNA Gene Sequencing

Total genomic DNA was extracted using the TIANamp Swab DNA Kit (TIANGEN, Beijing, China) after TS samples were vortexed with glass beads and TT/NPT samples were liquid nitrogen-ground. To eliminate any contamination caused by blood stains on the surface of the sterile tissues, surface decontamination of the samples was achieved by washing with PBS. Amplification of the 16S rRNA gene V3–4 region was performed with primers 338F and 806R (Huse et al., 2007) using TransStart Fastpfu DNA Polymerase (TransGen, Beijing, China). Cycling conditions were as follows: denaturation at 95°C for 2 min; 20 cycles of 45 s at 95°C, 30 s at 55°C, and 30 s at 72°C; and extension at 72°C for 5 min. PCR amplification was performed for each sample in triplicate, and the samples were purified using the AxyPrep DNA Gel Extraction kit (Axygen Scientific Inc., Union City, CA, United States) and assessed via spectrophotometry (QuantiFluor-ST, Promega). Equivalent pooled 16S rRNA PCR amplicons were sequenced on an Illumina MiSeq instrument with 2 × 300 bp paired-end sequencing.

### Bioinformatics and Statistical Analysis

Raw paired FASTQ files were imported into QIIME 2 (Bolyen et al., 2019), and the DADA2 plugin was used for further sequence quality control and chimera removal with default parameters. The merged high-quality sequences were used to produce amplicon sequence variants (ASVs) with 100% sequence identity. The taxonomic affiliation assignments were based on the Ribosomal Database Project (Cole et al., 2009) using default parameters (80% threshold). The representative sequence of each ASV was used as a query sequence to define species through BlastN against the Human Oral Microbiome Database (HOMD) RefSeq V15.22 (Escapa et al., 2018) and the online NCBI database with more than 99% identity and the highest total score.

Differences among the groups were assessed using analysis of similarities (ANOSIM). Microbiome functions were predicted using PICRUSt2 (Langille et al., 2013). Significant differences in taxa (species/ASVs, genus, and phylum) and microbiome functional profiles between TT, NPT, and TS groups were first determined by paired Wilcoxon signed-rank tests (Wilcoxon, 1946), and then the Benjamini–Hochberg false discovery rate (FDR) (Benjamini and Hochberg, 1995) was applied to adjust *p*-values for multiple tests in *R*. Significantly different features (FDR < 0.05) were further validated using linear discriminant analysis effect size (LEfSe) (Segata et al., 2011), and only features with an LDA score >2.0 were retained. The differences in taxa and microbiome functional profiles among different tumor sites and clinical stages were analyzed using LEfSe, and p values were adjusted using Benjamini–Hochberg FDR for multiple tests. The coefficient relationship between significantly changed taxa in different tumor sites or clinical stages and the corresponding microbial pathways of these groups were calculated using the Spearman correlation algorithm. The correlation parameters were set as coefficient >0.68 or <−0.68 and FDR < 0.05.

## Results

### Bacterial Populations of Different Oral Sites in Oral Squamous Cell Carcinoma

We collected 65 samples from 23 patients, including 23 TT, 23 NPT, and 19 saliva samples from the patients (Table 1). A total of 4,414,145 (23,645–174,760) high-quality 16S rRNA genes were obtained via high-throughput DNA sequencing. To normalize the data and avoid statistical bias, 23,645 16S rRNA genes from each sample were chosen to produce ASVs. A total of 4,164 ASVs were obtained. Good’s coverage was over 99.9% for each sample, suggesting that the sequencing depth was sufficient for microbiota investigation.

**TABLE 1:** Clinical characteristics of SCC subjects participating in the study.

Characteristics	Patients (*n* = 23)	
	
	Tissues (23)	Saliva (19)
Age (years)	61.9 ± 12.3	62.7 ± 12.1
**Sex**		
Male	11	10
Female	12	9
**Clinical stage**		
I–II	15	13
III–IV	8	6
Tumor sites		
Tongue	6	6
Bucca	3	3
Gingiva	11	7
Mouth floor	3	3
Tumor Grade*		
Well-differentiated	6	5
Moderately differentiated	14	12
Poorly differentiated	3	2
**Smoking status**		
Never	17	13
Former	3	3
Current	3	3
**Alcohol consumption**		
Never	17	13
Former	1	1
Current	5	5
**Oral hygiene**		
Fair	11	9
Bad	12	10
**Periodontitis status**		
No or mild	13	11
Moderate or severe	10	8

*The criteria of clinical stage are based on the AJCC Cancer Staging Manual. Values were displayed as mean ± SD.

**FIGURE 1:**
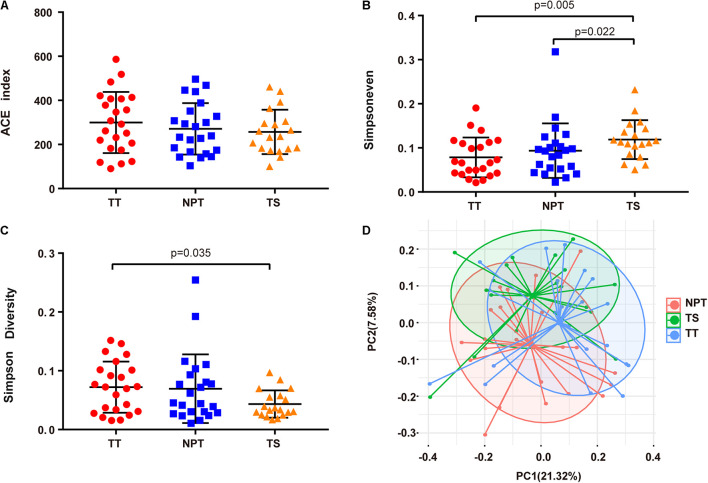
Comparison of alpha and beta diversity in TT, NPT, and TS groups. **(A)** ACE index, **(B)** Simpsoneven, **(C)** Simpson diversity, and **(D)** Principal Coordinate Analysis (PCoA) based on weighted UniFrac distance metrics.

In patients with OSCC, the evenness (Simpsoneven index) of the TS group was significantly different from that of the TT and NPT groups (Figures 1A–C and Supplementary Table 1). Principal coordinates analysis showed that the TS groups were significantly separated from the other groups (*P*_ANOSIM_ < 0.01, Figure 1D).

**FIGURE 2:**
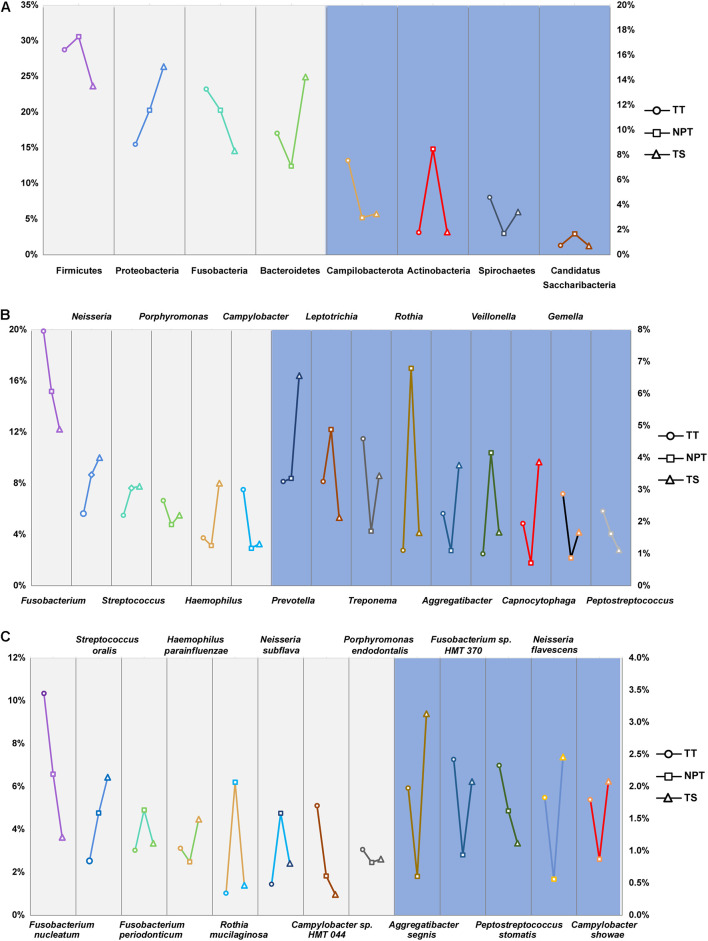
The proportion of major phyla **(A)**, genera **(B)**, and species **(C)** in the TT, NPT, and TS of OSCC patients. The relative abundance of taxa labeled in light gray was indicated by the left ordinate, and that labeled in light blue was indicated by the right ordinate.

A total of 22 phyla, 321 genera, and 869 species were identified in the oral samples, with the major taxa displayed in Figure 2. Firmicutes (27.93%), Proteobacteria (20.38%), Fusobacteria (19.67%), Bacteroidetes (17.73%), Campilobacterota (4.69%), Actinobacteria (4.17%), Spirochaetes (3.24%), and Candidatus Saccharibacteria (1.07%) were the eight major phyla identified in all samples (>1%). A total of 21 major genera (proportion >1%), such as *Fusobacterium* (15.99%), *Neisseria* (7.9%), *Streptococcus* (6.92%), *Porphyromonas* (5.66%), and *Haemophilus* (4.78%) were identified in the oral samples (Supplementary Table 2). Among these taxa, 12 phyla (>99.71% of all the samples), 115 genera (>97.98%), and 420 species (>97.49%) were consistently found in all samples.

### Bacterial Diversity Analysis of Tumor Tissue and Normal Paracancerous Tissue of Oral Squamous Cell Carcinoma Patients

**FIGURE 3:**
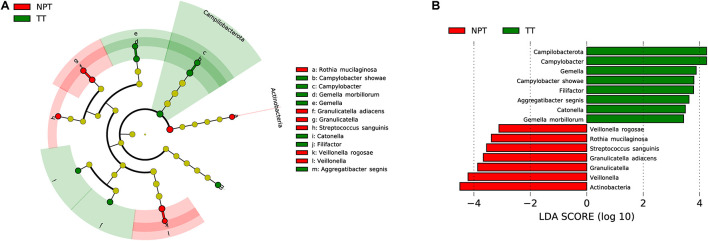
Comparative taxonomic profile of TT and NPT groups. **(A)** The cladogram showed differently enriched taxa. **(B)** LDA scores computed for differentially abundant taxa between TT and NPT groups (Log10 LDA scores >2). The phyla, genera, and species with significant richness differences (FDR < 0.05) between the two groups are shown.

The microbiota composition between TT (2,473 ASVs) and NPT (2,625 ASVs) from the same patients showed diversity (*P*_ANOSIM_ = 0.103, Figure 1D). The phylum Actinobacteria was significantly enriched in NPT, and the phylum Campilobacterota was significantly enriched in TT (Figure 3). The genera *Veillonella* and *Granulicatella* were highly enriched (proportion difference >0.5%) in NPT, and the genera *Campylobacter*, *Gemella*, *Filifactor*, and *Catonella* were highly enriched in TT (Figure 2). The species *Rothia mucilaginosa*, *G. adiacens*, *Streptococcus sanguinis*, and *Veillonella rogosae* were highly enriched (proportion difference >0.5%) in NPT, and *Aggregatibacter segnis*, *Campylobacter showae*, and *G. morbillorum* were highly enriched in TT (Figure 3).

Changes in the composition of the bacterial community altered the microbial functional profile. Through PICRUSt2 analysis, we found that only two pathways (Bifidobacterium shunt and heterolactic fermentation) were significantly enriched in TT compared with NPT (FDR < 0.05, proportions ratio >2) (Supplementary Table 3).

### Discrepancy Between the Tumor Tissue and Saliva Samples

**FIGURE 4:**
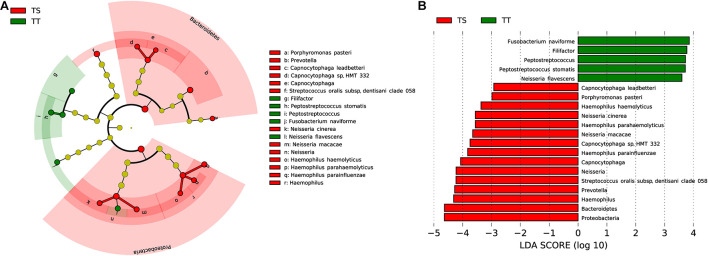
Comparative taxonomic profile of TT and TS groups. **(A)** The cladogram showed differently enriched taxa. **(B)** LDA scores computed for differentially abundant taxa between TT and TS groups (Log10 LDA scores >2). The phyla, genera, and species with significant richness differences (FDR < 0.05) between the two groups are shown.

The microbiota composition between the TT and TS of the same patient showed significant diversity (*P*_*ANOSIM*_ < 0.01, Figure 1D). The phyla Bacteroidetes and Proteobacteria were significantly enriched in TS samples (Figure 4). Four genera were highly enriched (proportion difference >0.5%) in TS samples, and the genera *Filifactor* and *Peptostreptococcus* were highly enriched in TT (Figure 4). Nine species were highly enriched (proportion difference >0.5%) in TS samples, including *Streptococcus oralis* and *Neisseria macacae*, and three species were enriched in TT (Figure 4), including *Neisseria flavescens*, *Fusobacterium naviforme*, and *S. stomatis*.

Thirty-seven and one pathways were separately enriched in TT and TS, respectively (FDR < 0.05, proportion ratio >2, Supplementary Table 4). Compared with TS, the function of aromatic compound degradation was highly enriched in TT, such as gallate degradation I (proportion ratio = 179).

### Discrepancy Between the Normal Paracancerous Tissue and Saliva Samples

**FIGURE 5:**
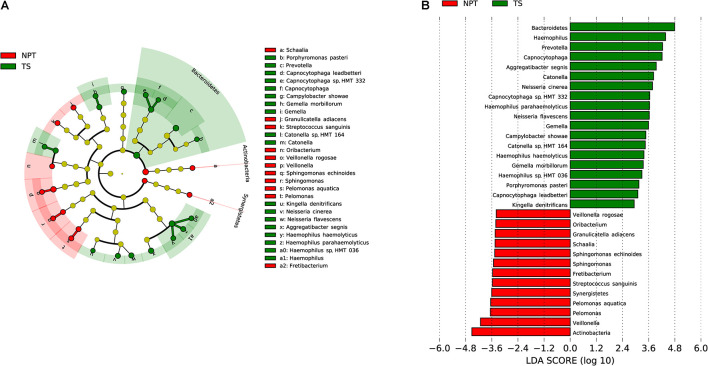
Comparative taxonomic profile of NPT and TS groups. **(A)** The cladogram showed differently enriched taxa. **(B)** LDA scores computed for differentially abundant taxa between TT and TS groups (Log10 LDA scores >2). The phyla, genera, and species with significant richness differences (FDR < 0.05) between the two groups are shown.

A comparison between NPT and TS samples also revealed significantly different taxa. Two phyla (Actinobacteria and Synergistetes), 6 genera, and 5 species were significantly enriched in NPT, while the phylum Bacteroidetes, 5 genera, and 13 species were significantly enriched in TS (Figure 5), including the three species highly enriched in TT relative to NPT.

Twenty-eight and two pathways were separately enriched in NPT and TS (FDR < 0.05, proportion ratio >2, Supplementary Table 5). Compared with TS, the function of amine and polyamine degradation was highly enriched in NPT, such as the superpathway of phenylethylamine degradation (proportion ratio = 343).

### Microbiota Diversity Analysis of Tissues at Different Tumor Sites

**FIGURE 6:**
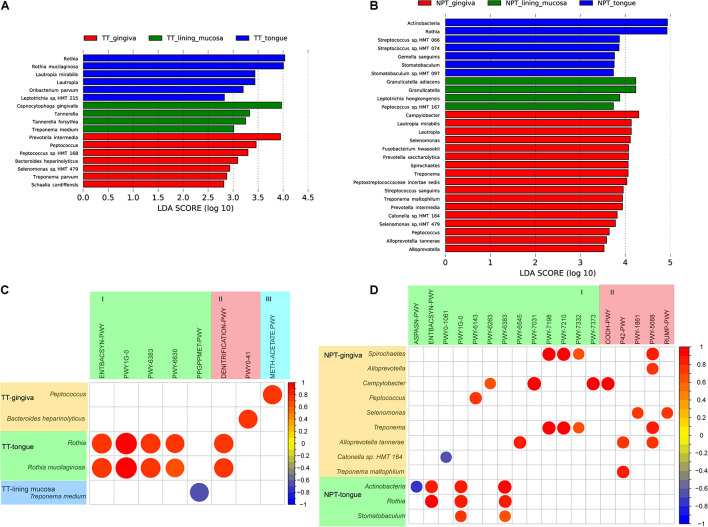
Comparative taxonomic profile and association with pathways of different tumor sites of OSCC. **(A)** LDA scores computed for differentially abundant taxa of TT among the tumor sites (Log10 LDA scores >2). **(B)** LDA scores computed for differentially abundant taxa of NPT among the tumor sites (Log10 LDA scores >2). **(C)** Correlation coefficient among different taxa and minipaths of TT samples among the different tumor sites. **(D)** Correlation coefficient among different taxa and minipaths of NPT samples among the different tumor sites. I, biosynthesis; II, degradation/utilization/assimilation; III, generation of precursor metabolites and energy.

All TT samples were collected from different sites involved in three types of oral mucosa, including the masticatory mucosa (gingiva), lining mucosa (bucca and mouth floor), and specialized mucosa (tongue). Four genera and 13 species were observed to be separately enriched in each of the three types of tumor mucosa (Figure 6A). *C. gingivalis, R, mucilaginosa*, and *P. intermedia* were significantly enriched in the lining mucosa, tongue, and gingiva, respectively. Although no significant differences were found in pathways among the three tumor sites, the correlation analysis of the pathway and species showed that the abundance changes of the three species were strongly related (*R* > 0.68) with seven different pathways (Figure 6C and Supplementary Table 6).

In addition, NPT samples from the three sites were more diverse, with two phyla (Actinobacteria and Synergistetes) and 10 genera and 16 species separately enriched in each of the three sites (Figure 6B). Most of these taxa were different from those observed in TT samples, indicating a different microenvironment between TTs and NPTs. The correlation analysis of the pathway and species showed that the abundance changes of the three species were strongly related (*R* > 0.68) with four different pathways (Figure 6D and Supplementary Table 7).

### Microbiota Diversity Analysis of Oral Cancer Stage

**FIGURE 7:**
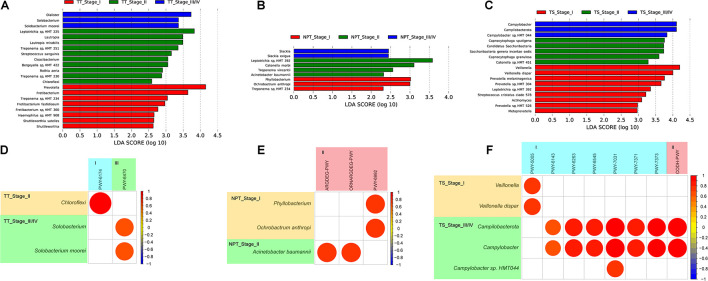
LDA scores computed for differentially abundant taxa among different clinical stages of TT **(A)**, NPT **(B)**, and TS **(C)** samples, and correlation coefficient among different species and minipaths in different clinical stages of TT **(D)**, NPT **(E)**, and TS **(F)** samples. I, biosynthesis; II, degradation/utilization/assimilation; III, generation of precursor metabolites and energy.

We further analyzed the relationship between oral microbiota and the clinical stage (I–IV) of oral cancer. In the early tumor stage (I/II), both TT and NPT had higher abundances of *Treponema* sp. and *Leptotrichia* sp., while *Prevotella* sp. and *Capnocytophaga* sp. were enriched in TS (Figures 7A–C). In the late tumor stage (III/IV), *Solobacterium moorei* and *Slackia exigua* were separately enriched in TT and NPT, while *Campylobacter* sp. *HMT 044* was enriched in TS (Figures 7A–C). The correlation analysis showed that only six pathways were positively associated with five significantly changed species in different clinical stages (Figures 7D,E and Supplementary Table 8); for example, *S. moorei* was positively related to peptidoglycan biosynthesis V.

## Discussion

Oral cancer is a highly complex, multifactorial disease associated with the dysbiosis of the oral microbiota. A comprehensive study of the oral microbiota is essential for understanding the pathogenesis of oral cancer. Previous studies have shown higher alpha diversity in saliva (Takahashi et al., 2019) and tissue biopsy samples (Zhang L. et al., 2019) of patients with oral cancer than that in healthy controls, and a recent study reported that TT has significantly lower richness and diversity than TS in patients with oral cancer (Zhang Z. et al., 2019). Here, we explored the oral microbiota composition of paired tumor/paracancerous tissues and saliva samples from patients with OSCC. Beta diversity analysis revealed a significant difference among TT, NPT, and TS samples, indicating that oral microbiota dysbiosis might be involved in the progression of OSCC (Figure 1).

Upon microbiota composition assessment between paired TT and NPT samples, in contrast with previous studies that reported increased Firmicutes abundance in TT compared to NPT (48 vs. 40%, respectively; *p* = 0.004) (Mukherjee et al., 2017), we did not find a significant change in the abundance of Firmicutes. The abundance of Actinobacteria was markedly decreased in TT (1.78% in TT vs. 8.48% in NPT, FDR < 0.01), coinciding with a previous report that the abundance of Actinobacteria decreased significantly with cancer progression (Yang et al., 2018), whereas the abundance of Campilobacterota was significantly increased in NPT (2.98% in NPT vs. 7.56% in TT, FDR < 0.05). At the species level, *R. mucilaginosa, F. periodonticum*, and *S. oralis* were among the most abundant taxa in the NPT, which is consistent with previous findings (Hooper et al., 2007; Moritani et al., 2015; Zhao et al., 2017). Furthermore, we identified *A. segnis*, *C. showae*, and *G. morbillorum* as significantly overrepresented species in both TT and TS (Figures 2, 3, 5). *A. segnis* is part of the oral microbiota, particularly in dental plaque (Norskov-Lauritsen and Kilian, 2006), and the aggregative nature of the pathogen in genus *Aggregatibacter* is crucial to its involvement in human disease (Norskov-Lauritsen et al., 2019). *C. showae*, a commensal species of the human oral cavity, has been associated with periodontitis (Lugonja et al., 2016), Crohn’s disease (Zhang et al., 2009), and colorectal cancer (Warren et al., 2013). *G. morbillorum* has been found to be relevant to the progression of periodontitis (Ai et al., 2017) and is highly associated with tumor site in patients with OSCC (Pushalkar et al., 2012). Therefore, these relatively abundant species may act as triggers for tumor initiation and progression.

Because the transient fluidity of surface TS differs from the stable environment of deep TT, the existing enriched oral microbiota could be distinguished in a site-specific manner. Interestingly, the comparison between TT and TS samples from the same patient revealed distinct microbial patterns associated with OSCC. We noted that the abundance of Proteobacteria (15.49% in TT vs. 26.40% in TS, FDR < 0.01) and Bacteroidetes (17.05% in TT vs. 24.95% in TS, FDR < 0.01) was remarkably higher in TS samples (Figure 4). Consistent with previous findings (Li et al., 2020), *Peptostreptococcus* and *Filifactor* were enriched in TT samples. At the species level, we confirmed that *F. naviforme*, *P. stomatis*, and *N. flavescens* were significantly enriched in TT. *F. naviforme* is known to be associated with the development and progression of OSCC (Fujiwara et al., 2020). Two reports have indicated that the abundance of *P. stomatis* is significantly higher in TT than in NPT (Pushalkar et al., 2012; Zhang L. et al., 2019). Additionally, nine species were observed in relatively higher proportions in TS samples, including *Neisseria cinerea* and *N. macacae*. As a high producer of the carcinogen acetaldehyde, a high abundance of *Neisseria* species in the saliva of patients with OSCC might play an important role in alcohol-related carcinogenesis (Muto et al., 2000). These findings indicate that unique pathogenic bacteria prefer inhabiting the inside/surrounding tumor sites, where the environment is different from the oral cavity.

The microbiota of different tumor sites in OSCC patients showed a homogenous population at a certain phylogenetic distance. Regarding the relative abundance of taxa, Zhang Z. et al. (2019) recently reported a greater abundance of *Prevotella*, *Acinetobacter*, *Pseudomonas*, and *Fusobacterium* in the tongue, oropharynx, gingiva, and bucca of patients with oral cancer. Here, our results revealed that in TTs, *Lautropia* and *Rothia* were enriched in the tongue, while *Peptococcus* and *Tannerella* were separately enriched in the gingiva and lining mucosa (Figure 6A). In NPTs, *Rothia* was enriched in the tongue, and *Lautropia* and *Peptococcus* were enriched in the gingiva (Figure 6B). The level of *P. intermedia* in the gingiva was significantly higher than that in the tongue and lining mucosa. As a periodontitis-related pathogen, *P. intermedia* is associated with an increased OSCC risk (Hsiao et al., 2018) and can release nuclease-degrading neutrophil extracellular traps (Doke et al., 2017). Increased abundance of *R. mucilaginosa* has been observed in oral leukoplakias from the tongue (Amer et al., 2017), and *R. mucilaginosa* can produce acetaldehyde comparable to that produced by *Neisseria mucosa* (Amer et al., 2020). *C. gingivalis* is thought to play an essential role in carcinogenesis (Karpinski, 2019) and may be a diagnostic indicator of OSCC (Mager et al., 2005). These findings indicate that the specific distribution of cancer-associated microbiota at different sites may be associated with the subsequent risk of OSCC.

The microbiota composition shown in the present study revealed differentially enriched species in TT, NPT, and TS samples of different OSCC stages (Figure 7). From the early stage to the late stage, the relative abundance of *S. moorei* in TT was significantly increased, and it is known to produce hydrogen sulfide and be associated with oral halitosis (Haraszthy et al., 2008). In addition, it is positively related to the pathway conferring pathogen beta-lactam resistance (Supplementary Table 8). We found that the relative abundances of *Veillonella dispar*, *Catonella morbi*, and *Leptotrichia* sp. *HMT 392* in both TT and TS microbiota were negatively correlated with the progression of OSCC, and that of *Campylobacter* sp. *HMT 044* was positively correlated. These differentially enriched species may be used as new biomarkers to predict OSCC progression.

At very low colonization levels, *P. gingivalis* can induce changes in oral commensal microbiota leading to periodontitis (Hajishengallis et al., 2011), which supports the “keystone pathogen” hypothesis (Hajishengallis et al., 2012). Therefore, we postulated that some low-abundance oral bacteria might also contribute to OSCC, although no significant changes were observed in the present study. To evaluate the possible functional changes caused by these low-abundance bacteria, we analyzed the functional relationship between species and pathways and found a correlation between 11 microbial species and 17 pathway abundances (Supplementary Tables 6–8). Since oral bacteria can interact through multiple pathways (Lamont et al., 2018), we postulated that significantly changed pathways, such as heterolactic fermentation, etc. (Supplementary Tables 3–5), might affect the growth of certain bacteria and lead to oral microbiota dysbiosis, thus finally inducing OSCC.

## Conclusion

In conclusion, the comparison analysis revealed that microbiota colonizing TT, NPT, and TS samples showed significant differences. The genus *Filifactor* and *Peptostreptococcus* showed the highest abundance in TT and the lowest abundance in TS samples, whereas *Neisseria* showed the opposite trend. At the species level, the abundance of *P. stomatis* decreased in the order of TT, NPT, and TS, while that of *S. oralis*, *N. macacae*, and *Capnocytophaga* sp. *HMT 332* increased in the same order. In addition, microbial communities showed variations in different tumor sites and clinical stages. The abundance of *P. intermedia* was profoundly higher in gingival tumor sites, whereas *C. gingivalis* and *R. mucilaginosa* were enriched in the lining mucosa and tongue. *S. moorei* in TT and *Campylobacter* sp. *strain HMT 044* in TS were positively correlated with the progression of OSCC. All these newly identified bacteria in the tissue surface or tumor microenvironment might be used as biomarkers to predict OSCC.

## Data Availability Statement

All the data generated or analyzed during this study are included in this published article and its Supplementary Material. The sequencing data from this study have been deposited in the National Omics Data Encyclopedia (NODE, https://www.biosino.org/node/index) under accession number OEX003132.

## Ethics Statement

The studies involving human participants were reviewed and approved by the Chinese Clinical Trial Registry (ChiCTR2000032543). The patients/participants provided their written informed consent to participate in this study.

## Author Contributions

TZ and HZ designed the study. KY, SZ, DZ, LH, and TZ collected all the saliva and tissue samples. YW and HZ performed the measurements and data analysis. KY, TZ, and HZ wrote and edited the manuscript. All authors read and approved the final manuscript.

## Conflict of Interest

The authors declare that the research was conducted in the absence of any commercial or financial relationships that could be construed as a potential conflict of interest.

## Publisher’s Note

All claims expressed in this article are solely those of the authors and do not necessarily represent those of their affiliated organizations, or those of the publisher, the editors and the reviewers. Any product that may be evaluated in this article, or claim that may be made by its manufacturer, is not guaranteed or endorsed by the publisher.

## Supplementary Material

Supplementary Table 1 The microbiota diversity evaluations of all groups.

Supplementary Table 2 Major abundant genera of all groups.

Supplementary Table 3 The different pathways between TT and NPT of OSCC patients.

Supplementary Table 4 The different pathways between TT and TS of OSCC patients.

Supplementary Table 5 The different pathways between NPT and TS of OSCC patients.

Supplementary Table 6 The correlation between significantly enriched TT species in different tumor sites and predicted microbial pathway (FDR < 0.05 and *R* > 0.68).

Supplementary Table 7 The correlation between significantly enriched NPT species in TT of different tumor grades and predicted microbial pathways (FDR < 0.05 and *R* > 0.68).

Supplementary Table 8 The correlation between significantly enriched species of different tumor clinical stages and predicted microbial pathways (FDR < 0.05 and *R* > 0.68).
