# Supplementary material for: Oral Microbiota Analysis of Tissue Pairs and Saliva Samples From Patients With Oral Squamous Cell Carcinoma – A Pilot Study
Source: Front Microbiol. 2021 Oct 12;12:719601. doi: 10.3389/fmicb.2021.719601 (PMC8546327; doi:10.3389/fmicb.2021.719601)
Supplement: Supplementary Table 1 — The microbiota diversity evaluations of all groups. [file Table_1.DOCX]

| **Group** | **ASVs** | **ACE** | **Simpsoneven** | **Simpson** | **Coverage** |
| --- | --- | --- | --- | --- | --- |
| Sample type |  |  |  |  |  |
| NPT | 2625 | 2644.83 | 0.037367 | 0.010195 | 99.99% |
| TT | 2473 | 2519.67 | 0.036493 | 0.011081 | 99.98% |
| TS | 1782 | 1802.29 | 0.058116 | 0.009656 | 99.99% |
| Tumor sites |  |  |  |  |  |
| lining mucosa | 992 | 1005.48 | 0.088494 | 0.011391 | 99.97% |
| Tongue | 950 | 966.0735 | 0.049496 | 0.021267 | 99.96% |
| Gingiva | 1659 | 1704.956 | 0.040881 | 0.014744 | 99.96% |
| **TT** |  |  |  |  |  |
| I | 1209 | 1236.242 | 0.060842 | 0.013595 | 99.95% |
| II | 1432 | 1477.853 | 0.039002 | 0.017905 | 99.95% |
| III/IV | 1191 | 1213.564 | 0.049443 | 0.016982 | 99.97% |
| NPT |  |  |  |  |  |
| I | 1132 | 1142.91 | 0.052451 | 0.016842 | 99.97% |
| II | 1547 | 1557.783 | 0.049956 | 0.01294 | 99.98% |
| III/IV | 1104 | 1116.524 | 0.043575 | 0.020787 | 99.98% |
| TS |  |  |  |  |  |
| I | 953 | 959.0589 | 0.088027 | 0.01192 | 99.99% |
| II | 981 | 996.1612 | 0.089191 | 0.011429 | 99.98% |
| III/IV | 820 | 834.0344 | 0.075818 | 0.016085 | 99.97% |
| Patients (TT + NPT +TS) | 4164 | 4212.452 | 0.029506 | 0.008139 | 99.99% |
