# Supplementary material for: Oral Microbiota Analysis of Tissue Pairs and Saliva Samples From Patients With Oral Squamous Cell Carcinoma – A Pilot Study
Source: Front Microbiol. 2021 Oct 12;12:719601. doi: 10.3389/fmicb.2021.719601 (PMC8546327; doi:10.3389/fmicb.2021.719601)
Supplement: Supplementary Table 2 — Major abundant genera of all groups. [file Table_2.DOCX]

| **Genus** | Total | **TT** | NPT | **TS** | **Lining  Mucosa** | **Tongue** | **Gingiva** | **TT-Stage** | | | **NPT-Stage** | | | **TS-Stage** | | |
| --- | --- | --- | --- | --- | --- | --- | --- | --- | --- | --- | --- | --- | --- | --- | --- | --- |
|  |  |  |  |  |  |  |  | **Ⅰ** | **Ⅱ** | **Ⅲ/Ⅳ** | **Ⅰ** | **Ⅱ** | **Ⅲ/Ⅳ** | **Ⅰ** | **Ⅱ** | **Ⅲ/Ⅳ** |
| Fusobacterium | 15.99% | 19.89% | 15.19% | 12.23% | 23.40% | 11.15% | 22.74% | 18.42% | 20.35% | 20.49% | 12.28% | 15.67% | 16.82% | 12.08% | 9.93% | 15.06% |
| Neisseria | 7.99% | 5.64% | 8.67% | 10.02% | 5.37% | 10.01% | 3.40% | 5.89% | 4.20% | 7.07% | 6.44% | 8.79% | 10.21% | 11.07% | 8.35% | 10.92% |
| Streptococcus | 6.92% | 5.50% | 7.63% | 7.78% | 4.60% | 10.51% | 3.26% | 6.06% | 6.38% | 4.10% | 9.39% | 6.19% | 7.94% | 8.97% | 5.53% | 9.20% |
| Porphyromonas | 5.66% | 6.66% | 4.78% | 5.52% | 5.67% | 4.73% | 8.25% | 10.89% | 4.07% | 6.40% | 8.01% | 4.51% | 2.65% | 5.40% | 6.16% | 4.89% |
| Haemophilus | 4.78% | 3.74% | 3.14% | 8.02% | 2.59% | 9.65% | 1.15% | 2.57% | 6.18% | 1.87% | 1.82% | 4.10% | 3.05% | 8.29% | 8.12% | 7.63% |
| Campylobacter | 4.65% | 7.51% | 2.93% | 3.27% | 6.51% | 8.66% | 7.44% | 3.43% | 8.74% | 9.20% | 1.32% | 5.16% | 1.62% | 1.66% | 3.70% | 4.39% |
| Prevotella | 4.26% | 3.26% | 3.35% | 6.57% | 2.00% | 3.28% | 3.93% | 4.23% | 1.76% | 4.22% | 3.94% | 2.52% | 3.85% | 9.31% | 5.63% | 4.92% |
| Leptotrichia | 3.50% | 3.26% | 4.88% | 2.13% | 4.77% | 3.29% | 2.42% | 3.54% | 4.61% | 1.53% | 7.31% | 4.66% | 3.31% | 3.45% | 1.39% | 1.68% |
| Rothia | 3.28% | 1.11% | 6.79% | 1.66% | 0.77% | 2.85% | 0.34% | 1.84% | 1.10% | 0.57% | 3.21% | 6.81% | 9.47% | 3.51% | 1.23% | 0.32% |
| Treponema | 3.24% | 4.59% | 1.71% | 3.44% | 4.40% | 1.98% | 6.12% | 5.43% | 5.78% | 2.64% | 1.57% | 2.68% | 0.71% | 2.69% | 3.81% | 3.77% |
| Veillonella | 2.31% | 1.00% | 4.15% | 1.68% | 0.52% | 2.29% | 0.56% | 1.07% | 1.52% | 0.36% | 5.00% | 3.71% | 4.01% | 4.30% | 0.50% | 0.42% |
| Aggregatibacter | 2.29% | 2.26% | 1.09% | 3.77% | 1.03% | 1.66% | 3.25% | 1.24% | 2.63% | 2.61% | 1.73% | 0.67% | 1.10% | 1.32% | 5.20% | 4.55% |
| Capnocytophaga | 2.07% | 1.95% | 0.71% | 3.87% | 3.09% | 0.78% | 1.96% | 1.95% | 2.45% | 1.37% | 0.51% | 0.82% | 0.75% | 3.53% | 6.30% | 1.38% |
| Gemella | 1.81% | 2.87% | 0.87% | 1.68% | 1.17% | 1.22% | 4.71% | 1.68% | 4.93% | 1.45% | 0.67% | 0.68% | 1.24% | 1.67% | 1.77% | 1.58% |
| Peptostreptococcus | 1.73% | 2.33% | 1.62% | 1.12% | 3.19% | 1.95% | 2.07% | 2.29% | 1.07% | 3.78% | 1.39% | 1.18% | 2.29% | 0.91% | 1.21% | 1.23% |
| Selenomonas | 1.41% | 0.91% | 2.25% | 1.00% | 0.61% | 1.14% | 0.94% | 0.52% | 0.72% | 1.41% | 0.91% | 1.89% | 3.68% | 0.60% | 1.33% | 1.00% |
| Parvimonas | 1.33% | 1.75% | 1.25% | 0.92% | 1.99% | 1.61% | 1.68% | 2.16% | 0.93% | 2.35% | 0.72% | 1.31% | 1.58% | 0.72% | 1.02% | 1.00% |
| Filifactor | 1.19% | 1.90% | 0.92% | 0.68% | 2.46% | 0.92% | 2.12% | 2.29% | 1.04% | 2.57% | 1.05% | 0.98% | 0.74% | 0.67% | 0.63% | 0.73% |
| Catonella | 1.16% | 1.13% | 0.59% | 1.89% | 1.27% | 0.77% | 1.26% | 1.20% | 0.91% | 1.33% | 0.48% | 0.78% | 0.47% | 1.68% | 2.43% | 1.49% |
| Saccharibacteria_genera _incertae_sedis | 1.07% | 0.75% | 1.68% | 0.72% | 0.77% | 0.34% | 0.96% | 0.79% | 0.43% | 1.08% | 0.70% | 2.31% | 1.72% | 0.67% | 1.30% | 0.10% |
| Granulicatella | 1.00% | 0.52% | 1.74% | 0.69% | 0.52% | 0.96% | 0.28% | 0.85% | 0.49% | 0.31% | 0.69% | 1.16% | 3.18% | 0.93% | 0.78% | 0.34% |
