# Supplementary material for: Oral Microbiota Analysis of Tissue Pairs and Saliva Samples From Patients With Oral Squamous Cell Carcinoma – A Pilot Study
Source: Front Microbiol. 2021 Oct 12;12:719601. doi: 10.3389/fmicb.2021.719601 (PMC8546327; doi:10.3389/fmicb.2021.719601)
Supplement: Supplementary Table 3 — The different pathways between TT and NPT of OSCC patients. [file Table_3.DOCX]

| **Class1** | **Class2** | **Pathway** | **TT (%)** | **NPT (%)** | **FDR** | **Ratio of  proportions** |
| --- | --- | --- | --- | --- | --- | --- |
| Degradation/Utilization/Assimilation | Carbohydrate Degradation | Bifidobacterium shunt | 0.0598 | 0.1343 | 0.0458 | 0.45 |
| Generation of Precursor Metabolites and Energy | Fermentation | heterolactic fermentation | 0.0510 | 0.1116 | 0.0458 | 0.46 |
