# Supplementary material for: Oral Microbiota Analysis of Tissue Pairs and Saliva Samples From Patients With Oral Squamous Cell Carcinoma – A Pilot Study
Source: Front Microbiol. 2021 Oct 12;12:719601. doi: 10.3389/fmicb.2021.719601 (PMC8546327; doi:10.3389/fmicb.2021.719601)
Supplement: Supplementary Table 4 — The different pathways between TT and TS of OSCC patients. [file Table_4.DOCX]

| **Class1** | **Class2** | **Pathway** | **TT (%)** | **TS (%)** | **FDR** | **Ratio of  proportions** |
| --- | --- | --- | --- | --- | --- | --- |
| Biosynthesis | Cell Structure Biosynthesis | teichoic acid (poly-glycerol) biosynthesis | 0.011911 | 0.002589 | 0.0451 | 4.60 |
| Biosynthesis | Amino Acid Biosynthesis | photorespiration | 0.025281 | 0.002386 | 0.0472 | 10.60 |
| Biosynthesis | Cofactor, Carrier, and Vitamin Biosynthesis | cob(II)yrinate a,c-diamide biosynthesis II (late cobalt incorporation) | 0.013056 | 0.000298 | 0.0443 | 43.77 |
| Biosynthesis | Cofactor, Carrier, and Vitamin Biosynthesis | adenosylcobalamin biosynthesis II (late cobalt incorporation) | 0.018231 | 0.000502 | 0.0346 | 36.30 |
| Biosynthesis | Cofactor, Carrier, and Vitamin Biosynthesis | adenosylcobalamin biosynthesis I (early cobalt insertion) | 0.047613 | 0.007026 | 0.0280 | 6.78 |
| Degradation/Utilization/ Assimilation | Alcohol Degradation | superpathway of glycerol degradation to 1,3-propanediol | 0.017951 | 0.005612 | 0.0206 | 3.20 |
| Degradation/Utilization/ Assimilation | Amine and Polyamine Degradation | allantoin degradation to glyoxylate III | 0.003066 | 0.000638 | 0.0455 | 4.80 |
| Degradation/Utilization/ Assimilation | Amine and Polyamine Degradation | allantoin degradation IV (anaerobic) | 0.001542 | 0.000325 | 0.0360 | 4.74 |
| Degradation/Utilization/ Assimilation | Amino Acid Degradation | superpathway of L-arginine, putrescine, and 4-aminobutanoate  degradation | 0.002223 | 0.000013 | 0.0388 | 173.25 |
| Degradation/Utilization/ Assimilation | Amino Acid Degradation | superpathway of L-arginine and L-ornithine degradation | 0.002223 | 0.000013 | 0.0378 | 173.25 |
| Degradation/Utilization/ Assimilation | Amino Acid Degradation | superpathway of L-threonine metabolism | 0.003026 | 0.000162 | 0.0440 | 18.63 |
| Degradation/Utilization/ Assimilation | Amino Acid Degradation | L-histidine degradation II | 0.010766 | 0.000626 | 0.0352 | 17.20 |
| Degradation/Utilization/ Assimilation | Aromatic Compound Degradation | gallate degradation I | 0.000696 | 0.000004 | 0.0444 | 179.43 |
| Degradation/Utilization/ Assimilation | Aromatic Compound Degradation | catechol degradation I (meta-cleavage pathway) | 0.009093 | 0.000304 | 0.0440 | 29.86 |
| Degradation/Utilization/ Assimilation | Aromatic Compound Degradation | superpathway of salicylate degradation | 0.003171 | 0.000142 | 0.0475 | 22.35 |
| Degradation/Utilization/ Assimilation | Aromatic Compound Degradation | catechol degradation II (meta-cleavage pathway) | 0.004190 | 0.000244 | 0.0357 | 17.17 |
| Degradation/Utilization/ Assimilation | Aromatic Compound Degradation | catechol degradation to 2-oxopent-4-enoate II | 0.003224 | 0.000231 | 0.0355 | 13.97 |
| Degradation/Utilization/ Assimilation | Aromatic Compound Degradation | protocatechuate degradation II (ortho-cleavage pathway) | 0.012208 | 0.001146 | 0.0366 | 10.66 |
| Degradation/Utilization/ Assimilation | Aromatic Compound Degradation | catechol degradation III (ortho-cleavage pathway) | 0.003639 | 0.000344 | 0.0479 | 10.58 |
| Degradation/Utilization/ Assimilation | Aromatic Compound Degradation | aromatic compounds degradation via &beta;-ketoadipate | 0.003639 | 0.000344 | 0.0470 | 10.58 |
| Degradation/Utilization/ Assimilation | Aromatic Compound Degradation | toluene degradation I (aerobic) (via o-cresol) | 0.022874 | 0.002599 | 0.0071 | 8.80 |
| Degradation/Utilization/ Assimilation | Aromatic Compound Degradation | toluene degradation II (aerobic) (via 4-methylcatechol) | 0.022874 | 0.002599 | 0.0142 | 8.80 |
| Degradation/Utilization/ Assimilation | C1 Compound Utilization and Assimilation | reductive acetyl coenzyme A pathway | 0.143333 | 0.065249 | 0.0478 | 2.20 |
| Degradation/Utilization/ Assimilation | Carboxylate Degradation | D-galactarate degradation I | 0.002554 | 0.000077 | 0.0445 | 33.32 |
| Degradation/Utilization/ Assimilation | Carboxylate Degradation | D-galacturonate degradation I | 0.075674 | 0.033897 | 0.0221 | 2.23 |
| Degradation/Utilization/ Assimilation | Carboxylate Degradation | superpathway of beta-D-glucuronide and D-glucuronate  degradation | 0.064622 | 0.030901 | 0.0282 | 2.09 |
| Degradation/Utilization/ Assimilation | Inorganic Nutrient Metabolism | methylphosphonate degradation I | 0.013253 | 0.001715 | 0.0300 | 7.73 |
| Degradation/Utilization/ Assimilation | Nucleoside and Nucleotide Degradation | adenosine nucleotides degradation II | 0.168443 | 0.069067 | 0.0203 | 2.44 |
| Degradation/Utilization/ Assimilation | Nucleoside and Nucleotide Degradation | guanosine nucleotides degradation III | 0.180233 | 0.083684 | 0.0367 | 2.15 |
| Degradation/Utilization/ Assimilation | Nucleoside and Nucleotide Degradation | purine nucleobases degradation I (anaerobic) | 0.162257 | 0.077084 | 0.0359 | 2.10 |
| Degradation/Utilization/ Assimilation | Nucleoside and Nucleotide Degradation | purine nucleotides degradation II (aerobic) | 0.210692 | 0.101471 | 0.0190 | 2.08 |
| Degradation/Utilization/ Assimilation | Secondary Metabolite Degradation | myo-inositol degradation I | 0.007165 | 0.002858 | 0.0349 | 2.51 |
| Generation of Precursor Metabolites and Energy | Respiration | methanogenesis from acetate | 0.012365 | 0.003663 | 0.0227 | 3.38 |
| Generation of Precursor Metabolites and Energy | Fermentation | glycerol degradation to butanol | 0.067044 | 0.023393 | 0.0401 | 2.87 |
| Generation of Precursor Metabolites and Energy |  | methylaspartate cycle | 0.010521 | 0.000384 | 0.0363 | 27.40 |
| Superpathways |  | superpathway of D-glucarate and D-galactarate degradation | 0.002554 | 0.000077 | 0.0438 | 33.32 |
| Superpathways |  | superpathway of hexuronide and hexuronate degradation | 0.057902 | 0.026413 | 0.0249 | 2.19 |
| Biosynthesis | Amine and Polyamine Biosynthesis | norspermidine biosynthesis | 0.009088 | 0.032963 | 0.0471 | 0.28 |
