# Supplementary material for: Oral Microbiota Analysis of Tissue Pairs and Saliva Samples From Patients With Oral Squamous Cell Carcinoma – A Pilot Study
Source: Front Microbiol. 2021 Oct 12;12:719601. doi: 10.3389/fmicb.2021.719601 (PMC8546327; doi:10.3389/fmicb.2021.719601)
Supplement: Supplementary Table 5 — The different pathways between NPT and TS of OSCC patients. [file Table_5.DOCX]

| **Class1** | **Class2** | **Pathway** | **NPT (%)** | **TS (%)** | **FDR** | **Ratio of  proportions** |
| --- | --- | --- | --- | --- | --- | --- |
| Biosynthesis | Amine and Polyamine Biosynthesis | norspermidine biosynthesis | 0.009225 | 0.032963 | 0.0394 | 0.28 |
| Biosynthesis | Cell Structure Biosynthesis | superpathway of (Kdo)2-lipid A biosynthesis | 0.121412 | 0.258204 | 0.0070 | 0.47 |
| Degradation/Utilization/Assimilation | Alcohol Degradation | superpathway of glycerol degradation to 1,3-propanediol | 0.013753 | 0.005612 | 0.0330 | 2.45 |
| Degradation/Utilization/Assimilation | Amine and Polyamine Degradation | allantoin degradation IV (anaerobic) | 0.002007 | 0.000325 | 0.0336 | 6.17 |
| Degradation/Utilization/Assimilation | Amine and Polyamine Degradation | allantoin degradation to glyoxylate III | 0.006087 | 0.000638 | 0.0316 | 9.54 |
| Degradation/Utilization/Assimilation | Amine and Polyamine Degradation | glycine betaine degradation I | 0.006188 | 0.000030 | 0.0315 | 207.22 |
| Degradation/Utilization/Assimilation | Amine and Polyamine Degradation | superpathway of phenylethylamine degradation | 0.002875 | 0.000008 | 0.0434 | 342.57 |
| Degradation/Utilization/Assimilation | Amino Acid Degradation | superpathway of L-threonine metabolism | 0.003492 | 0.000162 | 0.0267 | 21.50 |
| Degradation/Utilization/Assimilation | Aromatic Compound Degradation | toluene degradation I (aerobic) (via o-cresol) | 0.036995 | 0.002599 | 0.0044 | 14.23 |
| Degradation/Utilization/Assimilation | Aromatic Compound Degradation | toluene degradation II (aerobic) (via 4-methylcatechol) | 0.036995 | 0.002599 | 0.0046 | 14.23 |
| Degradation/Utilization/Assimilation | Aromatic Compound Degradation | catechol degradation II (meta-cleavage pathway) | 0.006768 | 0.000244 | 0.0385 | 27.73 |
| Degradation/Utilization/Assimilation | Aromatic Compound Degradation | catechol degradation I (meta-cleavage pathway) | 0.011877 | 0.000304 | 0.0077 | 39.00 |
| Degradation/Utilization/Assimilation | Carbohydrate Degradation | Bifidobacterium shunt | 0.134257 | 0.057868 | 0.0269 | 2.32 |
| Degradation/Utilization/Assimilation | Carboxylate Degradation | D-galacturonate degradation I | 0.078381 | 0.033897 | 0.0203 | 2.31 |
| Degradation/Utilization/Assimilation | Carboxylate Degradation | superpathway of &beta;-D-glucuronide and D-glucuronate  degradation | 0.072694 | 0.030901 | 0.0289 | 2.35 |
| Degradation/Utilization/Assimilation | Carboxylate Degradation | D-glucarate degradation I | 0.003973 | 0.000420 | 0.0391 | 9.45 |
| Degradation/Utilization/Assimilation | Carboxylate Degradation | D-galactarate degradation I | 0.003916 | 0.000077 | 0.0405 | 51.08 |
| Biosynthesis | Cell Structure Biosynthesis | teichoic acid (poly-glycerol) biosynthesis | 0.012576 | 0.002589 | 0.0029 | 4.86 |
| Biosynthesis | Cofactor, Carrier, and Vitamin Biosynthesis | adenosylcobalamin biosynthesis I (early cobalt insertion) | 0.049655 | 0.007026 | 0.0192 | 7.07 |
| Biosynthesis | Cofactor, Carrier, and Vitamin Biosynthesis | adenosylcobalamin biosynthesis II (late cobalt incorporation) | 0.027717 | 0.000502 | 0.0281 | 55.19 |
| Generation of Precursor Metabolites and Energy | Fermentation | heterolactic fermentation | 0.111619 | 0.049869 | 0.0282 | 2.24 |
| Degradation/Utilization/Assimilation | Nucleoside and Nucleotide Degradation | purine nucleobases degradation I (anaerobic) | 0.154655 | 0.077084 | 0.0288 | 2.01 |
| Degradation/Utilization/Assimilation | Nucleoside and Nucleotide Degradation | guanosine nucleotides degradation III | 0.176618 | 0.083684 | 0.0289 | 2.11 |
| Degradation/Utilization/Assimilation | Nucleoside and Nucleotide Degradation | adenosine nucleotides degradation II | 0.165570 | 0.069067 | 0.0283 | 2.40 |
| Degradation/Utilization/Assimilation | Secondary Metabolite Degradation | D-fructuronate degradation | 0.122392 | 0.059274 | 0.0250 | 2.06 |
| Degradation/Utilization/Assimilation | Secondary Metabolite Degradation | 4-deoxy-L-threo-hex-4-enopyranuronate degradation | 0.089852 | 0.040799 | 0.0281 | 2.20 |
| Degradation/Utilization/Assimilation | Secondary Metabolite Degradation | myo-, chiro- and scillo-inositol degradation | 0.020624 | 0.006359 | 0.0284 | 3.24 |
| Degradation/Utilization/Assimilation | Secondary Metabolite Degradation | myo-inositol degradation I | 0.015018 | 0.002858 | 0.0281 | 5.25 |
| Superpathways | | superpathway of hexuronide and hexuronate degradation | 0.061864 | 0.026413 | 0.0227 | 2.34 |
| Superpathways | | superpathway of D-glucarate and D-galactarate degradation | 0.003916 | 0.000077 | 0.0399 | 51.08 |
