# Supplementary material for: Oral Microbiota Analysis of Tissue Pairs and Saliva Samples From Patients With Oral Squamous Cell Carcinoma – A Pilot Study
Source: Front Microbiol. 2021 Oct 12;12:719601. doi: 10.3389/fmicb.2021.719601 (PMC8546327; doi:10.3389/fmicb.2021.719601)
Supplement: Supplementary Table 6 — The correlation between significantly enriched TT species in different tumor sites and predicted microbial pathway (FDR < 0.05 and R > 0.68). [file Table_6.DOCX]

| **Pathway** | **Species** | **Enriched in** | **Correlation coefficient** | **FDR** |
| --- | --- | --- | --- | --- |
| ppGpp biosynthesis | *Treponema medium* | lining mucosa | -0.70 | 0.036268 |
| allantoin degradation IV (anaerobic) | *Bacteroides heparinolyticus* | gingiva | 0.71 | 0.00021 |
| superpathway of L-tyrosine biosynthesis | *Rothia mucilaginosa* | tongue | 0.69 | 0.041947 |
| nitrate reduction I (denitrification) | *Rothia mucilaginosa* | tongue | 0.74 | 0.006061 |
| mono-trans, poly-cis decaprenyl phosphate biosynthesis | *Rothia mucilaginosa* | tongue | 0.77 | 0.000277 |
| enterobactin biosynthesis | *Rothia mucilaginosa* | tongue | 0.78 | 0.000112 |
| mycothiol biosynthesis | *Rothia mucilaginosa* | tongue | 0.91 | 0.000075 |
