# Supplementary material for: Oral Microbiota Analysis of Tissue Pairs and Saliva Samples From Patients With Oral Squamous Cell Carcinoma – A Pilot Study
Source: Front Microbiol. 2021 Oct 12;12:719601. doi: 10.3389/fmicb.2021.719601 (PMC8546327; doi:10.3389/fmicb.2021.719601)
Supplement: Supplementary Table 7 — The correlation between significantly enriched NPT species in TT of different tumor grades and predicted microbial pathways (FDR < 0.05 and R > 0.68). [file Table_7.DOCX]

| **Pathway** | **Species** | **Enriched in** | **Correlation coefficient** | **FDR** |
| --- | --- | --- | --- | --- |
| L-glutamate degradation VIII (to propanoate) | *Alloprevotella tannerae* | gingiva | 0.76 | 0.000174 |
| incomplete reductive TCA cycle | *Alloprevotella tannerae* | gingiva | 0.78 | 0.048009 |
| pyrimidine deoxyribonucleotides de novo biosynthesis III | *Alloprevotella tannerae* | gingiva | 0.83 | 0.026586 |
| superpathway of L-alanine biosynthesis | *Catonella* sp *HMT 164* | gingiva | -0.68 | 0.048517 |
| incomplete reductive TCA cycle | *Treponema maltophilum* | gingiva | 0.82 | 0.039674 |
