# Supplementary material for: Oral Microbiota Analysis of Tissue Pairs and Saliva Samples From Patients With Oral Squamous Cell Carcinoma – A Pilot Study
Source: Front Microbiol. 2021 Oct 12;12:719601. doi: 10.3389/fmicb.2021.719601 (PMC8546327; doi:10.3389/fmicb.2021.719601)
Supplement: Supplementary Table 8 — The correlation between significantly enriched species of different tumor clinical stages and predicted microbial pathways (FDR < 0.05 and R > 0.68). [file Table_8.DOCX]

| **Pathway** | **Species** | **Enriched in** | **Correlation coefficient** | **FDR** |
| --- | --- | --- | --- | --- |
| protein N-glycosylation (bacterial) | *Campylobacter* sp *HMT 044* | TS_Stage_III/IV | 0.79 | 0.023781 |
| biotin biosynthesis II | *Veillonella dispar* | TS_Stage_I | 0.78 | 0.000006 |
| superpathway of L-arginine, putrescine, and 4-aminobutanoate degradation | *Acinetobacter baumannii* | NTP_Stage_II | 0.76 | 0.003868751 |
| superpathway of L-arginine and L-ornithine degradation | *Acinetobacter baumannii* | NTP_Stage_II | 0.76 | 0.003868751 |
| 1,5-anhydrofructose degradation | *Ochrobactrum anthropi* | NTP_Stage_I | 0.74 | 6.48662E-07 |
| peptidoglycan biosynthesis V (beta-lactam resistance) | *Solobacterium moorei* | TT_Stage_III/IV | 0.68 | 0.0326828 |
